# Long-term ozone exposure and mortality in patients with chronic kidney disease: a large cohort study

**DOI:** 10.1186/s12882-024-03500-6

**Published:** 2024-02-28

**Authors:** Ejin Kim, Hyuk Huh, Yongwon Mo, Jae Yoon Park, Jiyun Jung, Hajeong Lee, Sejoong Kim, Dong Ki Kim, Yon Su Kim, Chun Soo Lim, Jung Pyo Lee, Yong Chul Kim, Ho Kim

**Affiliations:** 1 Institute of Health and Environment and Graduate School of Public Health, Seoul National University, Room 708, Building 220, Gwanak-Ro Gwanak-Gu, Seoul, 08826 Republic of Korea; 2 Department of Internal Medicine, Inje University Busan Paik Hospital, Busan, Republic of Korea; 3 Department of Landscape Architecture, Yeungnam University, Gyeongsan, Republic of Korea; 4 Department of Internal Medicine, Dongguk University Ilsan Hospital, Gyeonggi-Do, Republic of Korea; 5 Data Management and Statistics Institute, Dongguk University Ilsan Hospital, Ilsan, Republic of Korea; 6 Department of Internal Medicine, Seoul National University Hospital, Daehak-Ro, Jongno-Gu, 101 Seoul, Republic of Korea; 7 Kidney Research Institute, Seoul National University Hospital, Seoul, Republic of Korea; 8 Department of Medical Science, Seoul National University College of Medicine, Seoul, Republic of Korea; 9 Department of Internal Medicine, Seoul National University Boramae Medical Center, Seoul, Republic of Korea; 10 Department of Internal Medicine, Seoul National University Bundang Hospital, Seongnam, Republic of Korea; 11 Department of Biostatistics and Epidemiology, School of Public Health, Seoul National University, Seoul, Republic of Korea; 12 Department of Internal Medicine, Seoul National University College of Medicine, Seoul, Republic of Korea; 13 Department of Translational Medicine, Seoul National University College of Medicine, Seoul, Republic of Korea

**Keywords:** Chronic kidney disease, End stage renal disease, Mortality, Ozone

## Abstract

**Background:**

Epidemiologic studies on the effects of long-term exposure to ozone (O_3_) have shown inconclusive results. It is unclear whether to O_3_ has an effect on chronic kidney disease (CKD). We investigated the effects of O_3_ on mortality and renal outcome in CKD.

**Methods:**

We included 61,073 participants and applied Cox proportional hazards models to examine the effects of ozone on the risk of end-stage renal disease (ESRD) and mortality in a two-pollutants model adjusted for socioeconomic status. We calculated the concentration of ozone exposure one year before enrollment and used inverse distance weighting (IDW) for interpolation, where the exposure was evenly distributed.

**Results:**

In the single pollutant model, O_3_ was significantly associated with an increased risk of ESRD and all-cause mortality. Based on the O_3_ concentration from IDW interpolation, this moving O_3_ average was significantly associated with an increased risk of ESRD and all-cause mortality. In a two-pollutants model, even after we adjusted for other measured pollutants, nitrogen dioxide did not attenuate the result for O_3_. The hazard ratio (HR) value for the district-level assessment is 1.025 with a 95% confidence interval (CI) of 1.014–1.035, while for the point-level assessment, the HR value is 1.04 with a 95% CI of 1.035–1.045. The impact of ozone on ESRD, hazard ratio (HR) values are, 1.049(95%CI: 1.044–1.054) at the district unit and 1.04 (95%CI: 1.031–1.05) at the individual address of the exposure assessment. The ozone hazard ratio for all-cause mortality was 1.012 (95% confidence interval: 1.008–1.017) for administrative districts and 1.04 (95% confidence interval: 1.031–1.05) for individual addresses.

**Conclusions:**

This study suggests that long-term ambient O_3_ increases the risk of ESRD and mortality in CKD. The strategy to decrease O_3_ emissions will substantially benefit health and the environment.

**Supplementary Information:**

The online version contains supplementary material available at 10.1186/s12882-024-03500-6.

## Background

Air pollution has been recognized as a global health burden and there are concerns of preventable deaths due to air pollution [1, 2]. Some researchers reported that the risk of premature death from pollution was fifteen times higher than that of other factors including infectious diseases [3]. The most representative pollutants are particulate matter (PM_2.5_), nitrogen dioxide (NO_2_), carbon monoxide (CO), sulfur dioxide (SO_2_), and ozone (O_3_), emitted in the form of gas from vehicle exhaust or industrial production [4, 5]. Many epidemiologic studies have aimed to identify a causal relationship between air pollutants and mortality, disease progression. In an open cohort study conducted in the United States, an increase in O_3_ of 10 parts per billion (ppb) was associated with a 1.1% increase of all-cause mortality, and a significantly increased risk was observed below the national standard of 50 ppb [6]. However, Danish cohort study reported an inverse relationship between O_3_ concentrations and mortality risk [7]. These controversial results would be derived because the generation of ozone requires interaction with other exhaust gases including as nitrogen oxides (NOx) and carbon monoxide (CO) depending on sunlight.

Nitrogen oxides are mainly emitted from automobile exhaust gases, which get discharged into the atmosphere and combust to form nitrogen monoxide, which further combines with oxygen to form NO_2_. Then, NO_2_ gets photo-dissociated by ultraviolet radiation to separate into oxygen and nitrogen monoxide. This resultant oxygen atom combines with natural atmospheric oxygen to produce O_3_. Considering this mechanism of O_3_ generation, we used a NO_2_-adjusted two-pollutant model in this study. Many studies have been performed to determine the effects of O_3_ on cardiovascular and respiratory diseases in the general population [8–12]. However, study on the effects of O_3_ on the renal outcome and mortality in chronic kidney disease (CKD) is lacking. While it has been observed that there is a correlation between long-term exposure to O_3_ and the prevalence of CKD, as well as an inverse correlation with eGFR [13, 14], some experimental studies have reported that O_3_ may play a role in the treatment of kidney disease [15]. Therefore, further research is needed to determine whether long-term exposure to O_3_ affects the long-term prognosis of CKD. Therefore, we investigated the association between long-term O_3_ exposure and renal outcome, mortality risk of patients with CKD. To assess the consistency of these associations, we used a two-pollutant model to investigate the confounding of O_3_ measurement by NO_2_, an air pollutant and precursor of O_3_.

## Methods

### Study population

This study was based on collected data from a large-scale cohort of patients (*N* = 61,073) who visited one of the following hospitals: Seoul National University Hospital, Seoul National University Bundang Hospital, and Seoul National University Boramae Medical Center between January 2001 and December 2016. We enrolled patients who met the definition of CKD as outlined in the 2012 Kidney Disease Improving Global Outcomes (KDIGO) Clinical Practice Guideline for the Evaluation and Management of CKD report. Inclusion criteria comprised patients with functional and/or structural damage to the kidneys lasting more than 3 months, while those with less than three months of observation were excluded. To investigate effect of ozone on individuals, data from a nationwide and district disease surveillance, collected at the high spatial resolution, was included.

For the purpose of allocating personal exposure, all participants were included, regardless of their place of residence, by utilizing the administrative district unit of personal address. In order to allocate exposure at a high resolution, the user's personal address was transformed into latitude and longitude coordinates. Additionally, a weight was assigned that varied inversely with the distance to the observatory. Exposure allocation was only directed towards residents of Seoul.

### Exposure assessment and assignment

We obtained hourly O_3_ concentrations from 533 air quality monitors at the Korea Environmental Corporation between 2001 and 2016. We defined ozone concentration in terms of moving 8-h averages, i.e., the average value of the 8-h maximum ozone concentration on a given day. We calculated the moving 8-h averages between 12:00 AM–8:00 AM on one day and 4:00 PM–12:00 AM on the next day. We divided personal exposure into two distinct methods. Initially, we assigned it to the city, county, and district administrative entities according to the individual's residence. Furthermore, by utilizing the complete personal address data, the address was transformed into latitude and longitude coordinates. Subsequently, personal exposure was determined using the Inverse Distance Weighting (IDW) technique, which relies on the address details of neighboring observatories centered on the specific location. We used mean concentration aggregation data based on each district to calculate the individual residence-based exposure assignment. Geographic Information System (GIS)-based pollution mapping often uses interpolation techniques, such as inverse-distance weighting (IDW), kriging, and land-use regression modeling [16]. We assigned ozone concentrations to the home addresses of our patients using the nearest monitor and IDW. For each day, we assigned a concentration from the operational monitor closest to the address of interest. Since interpolation is based on observing data from the monitoring site, we extracted the ozone concentration in Seoul, where monitors are distributed across each of the 25 administrative districts. Subsequently, we computed the monthly average exposure for each patient during their follow-up period.

### Outcomes and covariates

The outcome of study was cause-specific mortality and the incidence of ESRD. Death certificate data were obtained from the Korea National Statistical Office. ESRD were defined as patients who had a confirmed diagnostic code for ESRD, or had a prescription for dialysis, and had a history of arteriovenous fistula procedure and a catheter inserted for peritoneal dialysis and, kidney transplantation. Baseline information was collected at the time of enrollment. The estimated glomerular filtration rate (eGFR) was used to determine the stage of CKD. We used the modification of diet in the renal disease equation (MDRD) to estimate GFR. Individuals with a systolic blood pressure ≥ 140 mmHg or diastolic blood pressure ≥ 90 mmHg upon measurement, or a confirmed diagnostic code for hypertension (HTN), or a prescription for an antihypertensive drug, were classified as having HTN. Individuals with a diagnostic code for diabetes mellitus (DM) or a prescription for an antidiabetic drug including RAS blockers, beta-blockers, calcium channel blockers, alpha-blockers, and thiazide diuretics, were classified as having DM. The diagnostic codes for cardiovascular disease (CVD) (I00-I99), cancer (C00-C97), respiratory disease (J00-J99), stroke (I20-I22, I24-I25), and chronic obstructive pulmonary disease (COPD) (J44) were used to determine cause-specific mortality.

### Statistical analyses

The Chi-square test of independence was applied to check for an association between the ozone concentration levels and risk factors (categorical variables). The average numbers of morbidities between patient groups based on ozone concentration were compared using one-way analysis of variance (ANOVA) when appropriate (level of significance was set at 0.05). We applied Cox Proportional Hazards Models to examine associations between mean O_3_ concentrations, ESRD, and age-related mortality using an underlying time scale that followed each patient from their date of inclusion in the cohort until the date of death or December 2015. To assess ESRD as the outcome, we considered patients from their enrollment date until the date of death or the last follow-up date. Several sensitivity analyses were carried out to assess the robustness of the results. First, individual ozone concentration exposure was assessed by assigning it to each administrative district, and individual address latitude and longitude coordinates. Second, in order to rule out the potential confounding effects of other significant air pollutants, we conducted two-pollutant models that included NO2 pollution in the main effect models. Third, the analysis was conducted after individuals with deteriorating CKD conditions that contributed to ESRD or mortality were excluded. The supplementary material contains results of ozone effects in people with less severe illness (Table S1).

We used the Arc GIS 10.0 ESRI software and the ‘pspline’ function of ‘coxph’ to obtain smoothed spline predictions using the statistical software R version (3.6.0). All other analyses were conducted in SAS version 9.4 (SAS Institute Inc., Cary, NC).

## Results

### Baseline characteristics

We included 56,470 participants across three hospitals aged 58.37 ± 17.37 years having the estimated glomerular filtration rate (eGFR) of 61.07 ± 29.92 mL/min/1.73m^2^. There were 29,961 men (48.82%). 23.06% were diagnosed with diabetic mellitus and 21.85% with hypertension. The proportion of participants with CKD stage 3 was 29.42%, and advanced CKD with less than GFR 30 mL/min/1.73m^2^ was 16.79% (Table 1).

**Table 1:** Baseline characteristics of the study population according to exposure to ozone

Variables	Category	Total	Ozone (ppb)				***p*-value**
			Q1 [ 5 -27.54)	Q2 [ 27.54–31.78)	Q3 [ 31.78–35.38)	Q4 [ 35.38–199.63)	
	N	56,470	14,117	14,117	14,118	14,118	
	Missing	4603					
**Outcomes**							
End-stage renal disease (ESRD)	N	5957	1513	1432	1478	1534	0.2144
Follow up duration (ESRD)	Mean ± SD	71.64 ± 52.96	107.85 ± 59.82	83.45 ± 50.31	56.37 ± 40.65	42.03 ± 36.38	
All-cause mortality	N	6768	2193	1865	1523	1182	< 0.001
Follow up duration (Mortality)	Mean ± SD	82.12 ± 51.67	119.93 ± 54.20	99.04 ± 44.89	64.69 ± 39.70	47.21 ± 36.61	
NO2	Mean ± SD	33.60 ± 6.54	39.29 ± 15.32	36.75 ± 14.02	34.82 ± 14.12	28.17 ± 13.80	< 0.001*
PM10	Mean ± SD	56.79 ± 9.05	60.24 ± 42.25	58.61 ± 33.41	54.06 ± 32.98	50.89 ± 29.92	< 0.001*
Age	Mean ± SD	58.37 ± 17.37	62.00 ± 16.82	57.87 ± 17.38	58.17 ± 17.61	58.45 ± 17.22	< 0.001*
Sex		56,470					< 0.001
	male	29,961(48.82)	6673(11.82)	6693(11.85)	6866(12.16)	7335(12.99)	
	female	28,903(51.18)	7444(13.81)	7424(13.15)	7252(12.84)	6783(12.01)	
Education level (%)		8423					< 0.001
	Elementary	3133 (15.59)	1532 (6.54)	725 (3.10)	809 (3.45)	828 (3.53)	
	Middle-high	9105(46.36)	4131 (17.64)	2079 (8.88)	2228 (9.51)	2539 (10.84)	
	College, University	7403 (37.69)	3595 (15.35)	1755 (7.49)	1553 (6.63)	1650 (7.04)	
	missing	41,432					
Income level		19,641					
	Low	1668(19.80)	348(4.13)	394(4.68)	445(5.28)	481(5.71)	0.307
	High	6755(80.20)	1553(18.44)	1558(18.50)	1776(21.09)	1868(27.89)	
	missing	52,650					
BMI		56,470	23.25 ± 3.54	23.62 ± 3.75	23.58 ± 3.71	23.80 ± 3.82	< 0.001
	BMI < 18.5	21,019(37.22)	6082(10.77)	5374(9.52)	5009(8.87)	4554(8.06)	
	18.5 ≤ BMI < 23	14,477(25.64)	3545(6.28)	3569(6.32)	3650(6.46)	3713(6.58)	
	23 ≤ BMI < 25	8526(15.10)	1936(3.43)	2118(3.75)	2222(3.93)	2250(3.98)	
	25 ≤ BMI	12,448(22.04)	2554(4.52)	3056(5.41)	3237(5.73)	3601(6.38)	
Diabetic Mellitus	No	43,450(76.94)	11,001(19.48)	10,939(19.37)	10,799(19.12)	10,711(18.97)	< 0.001
	Yes	13,020(23.06)	3116(5.52)	3178(5.63)	3319(5.88)	3407(6.03)	
	missing	4603					
Hypertension	No	44,132(78.15)	11,861(21)	10,710(18.97)	10,564(18.71)	10,997(19.47)	< 0.001
	Yes	12,338(21.85)	2256(4)	3407(6.03)	3554(6.29)	3121(5.53)	
	missing	4603					
e-GFR(mL/min/1.73 m^2^)		61.07 ± 29.92	61.32 ± 27.35	61.42 ± 28.57	61.74 ± 30.39	60.32 ± 32.63	< 0.001*
CKD stage	< 30	9481(16.79)	2142(3.79)	2213(3.92)	2374(4.2)	2752(4.87)	< 0.001
	30–59	16,616(29.42)	3872(6.86)	4102(7.26)	4238(7.5)	4404(7.8)	
	60–89	21,702(38.43)	6375(11.29)	5789(10.25)	5110(9..05)	4428(7.84)	
	89 <	8671(15.36)	1728(3.06)	2013(3.56)	2396(4.24)	2534(4.49)	
	missing	4603					
Beds in medical facilities per 1,000 people		8.75 ± 3.51	9.31 ± 3.83	8.68 ± 3.24	8.19 ± 2.66	9.03 ± 4.29	< 0.001*
Financial independence		52.17 ± 15.97	54.02 ± 15.76	54.93 ± 14.90	53.23 ± 15.4	45.40 ± 16.41	< 0.001*

*NO*_*2*_ nitrogen dioxide, *PM*_*10*_ particulate matter, *CKD* chronic kidney disease, *eGFR* estimated glomerular filtration rate

^*^Anova one-way analysis

Our data is not independent of gender, education level, BMI, diagnosis of diabetes, diagnostic of hypertension, and CKD stage level, as indicated by the ozone concentration level (*P* < 0.001). During the study period, out of a total of 56,470 patients with chronic kidney disease (CKD), there were 5,957 cases of end-stage renal disease (ESRD) and 6,768 deaths were observed. The follow-up period for end-stage renal disease (ESRD) was 4,017,437.27 months (mean ± standard deviation: 71.64 ± 52.96), while the follow-up period for death was 4,576,686.23 months (mean ± sd: 82.12 ± 51.67). It is not possible to conclude that deaths are independent of ozone concentration level (*p*-value < 0.001), while ESRD was unable to reject the null hypothesis that there is no link depending on ozone concentration (*p*-value = 0.2144). (Table 1).

### Ozone concentration during study period

**Fig. 1:**
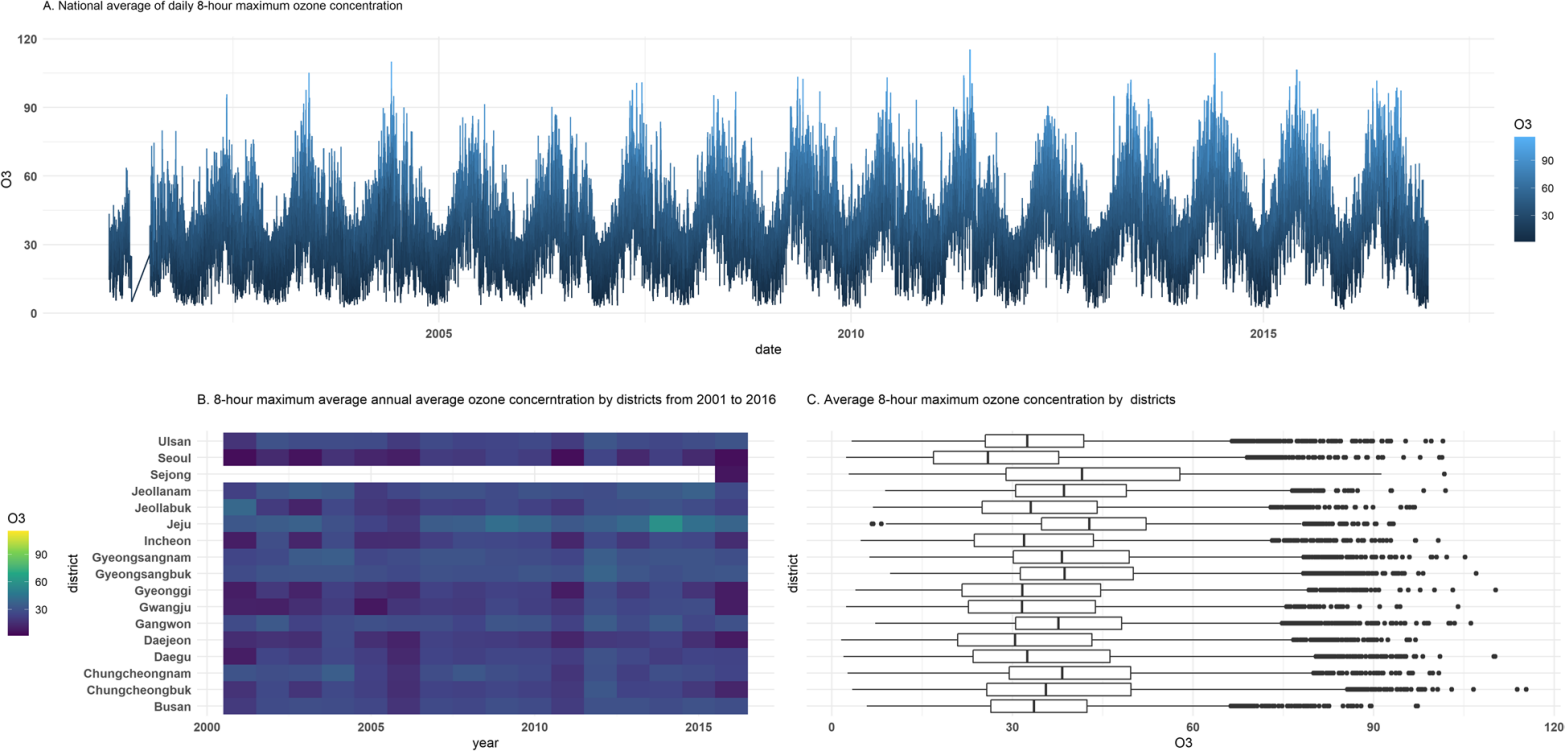
Moving average of the 8-h maximum ozone concentration for 365 days in the districts in 2001–2017

During the study period, the mean concentrations of O_3_ were 31.2 ppb. The time series plot showed the national average daily 8-h maximum O_3_ concentration; there were days when air quality standard O_3_ concentrations (60 ppb for each 8-h average) were exceeded (Fig. 1A). The heat map plot shows that the O_3_ concentration in Jeju increased more recently compared to other districts (Fig. 1B). In the box plot, except for the Sejong region, as can be seen from the B plot, it was evident that the average O_3_ concentration value in the Jeju region was high. On the other hand, it was found that the O_3_ concentration value in Seoul was lower than in other districts during the study period (Fig. 1C).

### Association between ozone concentration and outcomes

A total of 6,768 deaths occurred during the study period. We found that the moving O_3_ average of the districts for 365 days was significantly associated with an increased risk of ESRD (hazard ratio [HR] 1.034; 95% confidence interval [CI], 1.031–1.036) and all-cause mortality (HR, 1.02; 95% CI, 1.018–1.023) (Table 2, Model). Based on the O_3_ concentration from IDW interpolation, this moving O_3_ average was significantly associated with an increased risk of ESRD (HR, 1.019; 95% CI, 1.011–1.026) and all-cause mortality (HR, 1.047; 95% CI, 1.041–1.054). Even after we adjusted for other measured pollutants, NO_2_ did not attenuate the results for O_3_ (Table 2, Model 2). Additionally, we examined the nonlinearity of this association, but this was not statistically significant. However, the estimated exposure–response curves for O_3_ according to outcomes were almost linear (Figure S1).

**Table 2:** Association of annual mean maximum daily 8-h O3 concentrations from the previous year (1-year moving average) with ESRD and all-cause mortality in participants with CKD

Outcomes	End-stage renal disease (ESRD)						All-cause morality					
Exposure assessments	Districts			Points			Districts			Points		
	HR	95% CI		HR	95% CI		HR	95% CI		HR	95% CI	
Model												
Age	1	0.999	1.002	1	0.998	1.003	1.068	1.066	1.07	1.06	1.057	1.063
Male	1.406	1.349	1.465	1.365	1.264	1.475	1.72	1.651	1.792	1.827	1.706	1.957
Hypertension	0.958	0.915	1.003	0.863	0.785	0.949	1.141	1.093	1.192	1.594	1.483	1.713
Diabetic Mellitus	2.629	2.517	2.747	2.371	2.187	2.571	1.524	1.462	1.588	1.409	1.315	1.509
BMI < 18.5	0.266	0.25	0.282	0.289	0.257	0.324	0.745	0.709	0.782	0.879	0.812	0.952
23 ≤ BMI < 25	0.628	0.593	0.665	0.634	0.57	0.705	0.605	0.57	0.642	0.571	0.517	0.63
25 $\le$ BMI	0.552	0.524	0.581	0.578	0.524	0.636	0.526	0.496	0.557	0.419	0.379	0.463
O3 (ppb)	1.034	1.031	1.036	1.019	1.011	1.026	1.02	1.018	1.023	1.047	1.041	1.054
Model1												
Age	0.986	0.985	0.988	0.986	0.984	0.989	1.059	1.057	1.06	1.051	1.048	1.054
Male	1.214	1.165	1.265	1.212	1.122	1.309	1.608	1.544	1.675	1.701	1.589	1.821
Hypertension	0.963	0.92	1.008	0.8	0.728	0.879	1.142	1.094	1.193	1.576	1.467	1.693
Diabetic Mellitus	1.71	1.638	1.785	1.67	1.543	1.808	1.304	1.251	1.359	1.236	1.154	1.324
BMI < 18.5	0.458	0.43	0.487	0.436	0.388	0.491	0.951	0.906	0.999	1.075	0.992	1.165
23 ≤ BMI < 25	0.842	0.795	0.891	0.879	0.79	0.978	0.677	0.637	0.719	0.642	0.581	0.709
25 $\le$ BMI	0.799	0.759	0.842	0.889	0.806	0.981	0.608	0.574	0.644	0.481	0.435	0.533
e-GFR(mL/min/1.73 m^2^)	0.929	0.928	0.931	0.927	0.925	0.929	0.972	0.971	0.973	0.974	0.973	0.976
O3 (ppb)	1.025	1.023	1.028	1.02	1.013	1.028	1.016	1.014	1.019	1.046	1.039	1.053
Model2												
Age	0.987	0.986	0.988	0.987	0.984	0.99	1.06	1.058	1.061	1.05	1.047	1.053
Male	1.216	1.165	1.269	1.193	1.097	1.297	1.591	1.524	1.66	1.671	1.553	1.798
Hypertension	0.929	0.887	0.974	0.778	0.705	0.858	1.077	1.03	1.126	1.537	1.427	1.656
Diabetic Mellitus	1.683	1.61	1.76	1.673	1.535	1.823	1.269	1.215	1.325	1.165	1.082	1.255
BMI < 18.5	0.489	0.458	0.522	0.449	0.394	0.512	0.915	0.869	0.964	1.017	0.932	1.109
23 ≤ BMI < 25	0.845	0.796	0.896	0.892	0.794	1.003	0.665	0.625	0.708	0.632	0.569	0.702
25 $\le$ BMI	0.778	0.737	0.822	0.866	0.779	0.962	0.599	0.564	0.636	0.458	0.411	0.509
e-GFR(mL/min/1.73 m^2^)	0.927	0.926	0.928	0.922	0.92	0.925	0.972	0.971	0.973	0.975	0.973	0.976
O3 (ppb)	1.049	1.044	1.054	1.023	1.013	1.033	1.015	1.01	1.019	1.048	1.04	1.057
NO2(ppb)	1.017	1.013	1.02	0.992	0.983	1.001	1.006	1.003	1.01	1.019	1.012	1.026
Model3												
Age	0.987	0.986	0.989	0.987	0.984	0.989	1.06	1.058	1.062	1.051	1.048	1.054
Male	1.219	1.168	1.272	1.191	1.096	1.295	1.594	1.527	1.664	1.678	1.56	1.806
Hypertension	0.926	0.883	0.971	0.786	0.712	0.867	1.074	1.027	1.124	1.538	1.428	1.657
Diabetic Mellitus	1.676	1.603	1.753	1.675	1.537	1.825	1.269	1.215	1.325	1.168	1.085	1.258
BMI < 18.5	0.493	0.462	0.526	0.452	0.396	0.514	0.912	0.865	0.961	1.014	0.929	1.106
23 ≤ BMI < 25	0.843	0.795	0.895	0.893	0.794	1.003	0.662	0.622	0.705	0.633	0.57	0.702
25 $\le$ BMI	0.778	0.737	0.821	0.865	0.779	0.962	0.601	0.566	0.638	0.461	0.414	0.513
e-GFR(mL/min/1.73 m^2^)	0.927	0.926	0.928	0.922	0.92	0.925	0.972	0.971	0.973	0.975	0.973	0.977
O3 (ppb)	1.049	1.044	1.054	1.025	1.014	1.035	1.012	1.008	1.017	1.04	1.031	1.05
NO2(ppb)	1.016	1.012	1.02	0.991	0.982	1	1.004	1	1.008	1.025	1.018	1.033
Beds in medical facilities per 1,000 people	1.003	0.997	1.01	1.012	1	1.025	0.979	0.973	0.985	0.992	0.982	1.002
Financial independence	1.001	1	1.003	1	0.997	1.003	0.999	0.998	1.001	0.995	0.992	0.998

*ESRD* end stage renal disease, *O*_*3*_ ozone, *NO*_*2*_ nitrogen dioxide, *HR* hazard ratio, *95% CI* 95% confidence intervals

In the district’s allocation model, the impact of ozone on the hazard ratio of ESRD was 1.034 (95%CI:1.031–1.036), while in the model with point allocation, it was 1.019 (95%CI:1.011–1.026). In the model that additionally adjusted for eGFR, the HRs for ESRD were 1.025 (95%CI:1.023–1.028) and 1.02 (95%CI:1.013–1.028), respectively, and the HRs for death were 1.016 (95%CI:1.014–1.019) and 1.046 (95%CI:1.039–1.053). In the final model, the HR of ozone on ESRD was estimated to be 1.049 (95%CI:1.044–1.054) and 1.025 (95%CI:1.014–1.035), respectively, depending on exposure allocation, and for death, the HR was 1.012 (95%CI:1.008–1.017) and the HR was 1.04 (95%CI:1.031- 1.05) respectively (Table 2, Model 3). Subgroup analysis revealed that the impact of ozone on end-stage renal disease (ESRD) varied depending on factors such as age group, body mass index (BMI), diabetes mellitus (DM), and chronic kidney disease (CKD) stage. Furthermore, when exposure allocation was based on personal residence address, significant differences in the effects of ozone were observed among subgroups based on BMI, hypertension, and CKD. The variables that showed a significant impact on mortality in the district’s allocation model were age, BMI, DM (diabetes mellitus), and CKD stage (chronic kidney disease stage). In the points allocation model, the significant variables for mortality were BMI, hypertension, and CKD stage (Table S2).

**Fig. 2:**
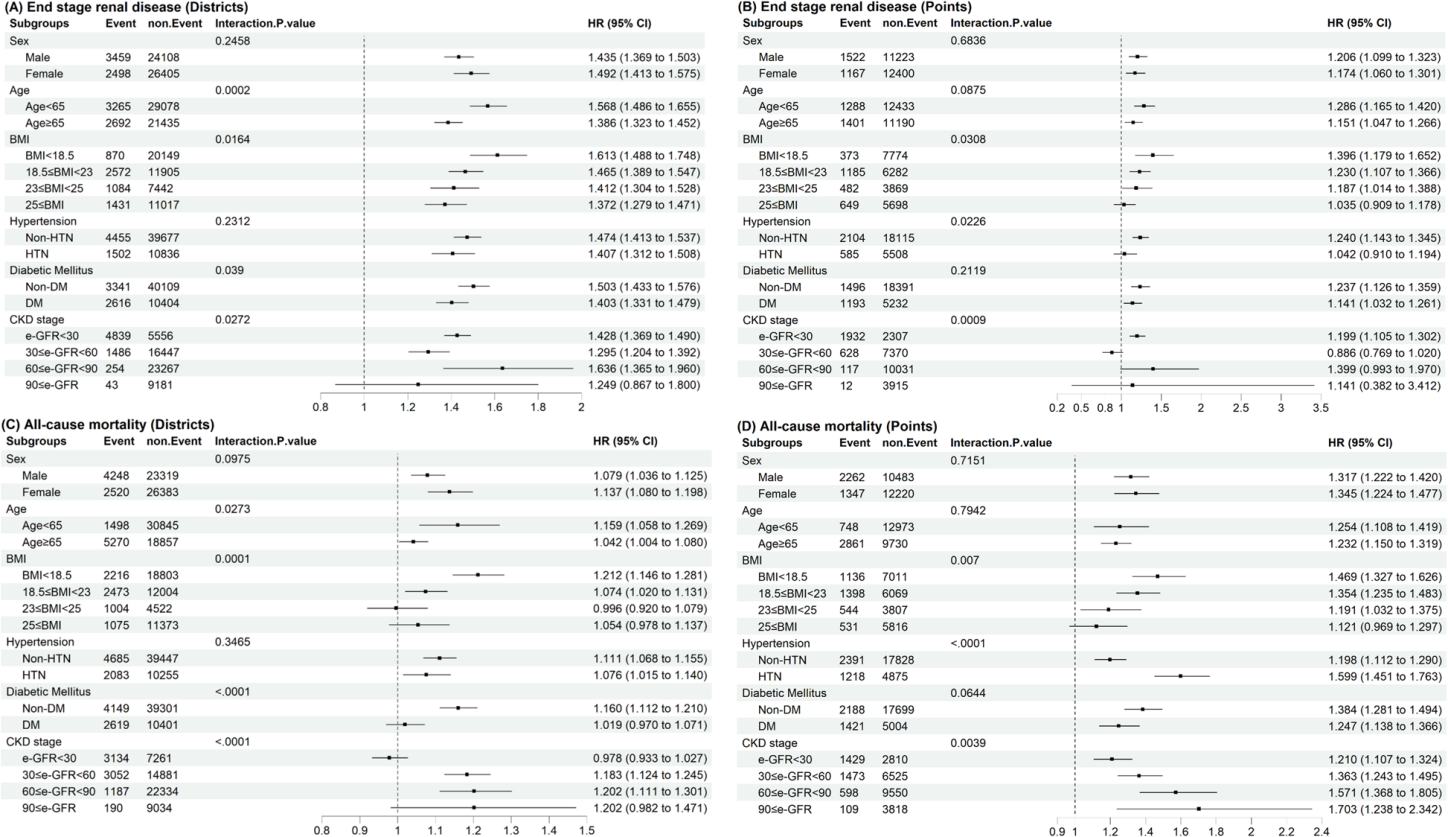
Hazard ratios for ozone concentration IQR unit change, for ESRD and all causes of death by subgroups

Figure 2 shows the calculated HR values for ESRD and all deaths when the ozone concentration in each subgroup changes IQR in the SUBGROUP analysis model. The hazard ratio (HR) for those under 65 years old was 1.568 (95% confidence interval [CI]: 1.468–1.655). For the group with a body mass index (BMI) over 25, the HR was 1.372 (95% CI: 1.279–1.471). The non-diabetic group had an HR of 1.503 (95% CI: 1.433–1.576). Lastly, for individuals with an estimated glomerular filtration rate (eGFR) between 60 and 90, the HR was 1.636 (95% CI: 1.365–1.960) (Fig. 2A). Figure 2B illustrates the significant influence of ozone, with a value of 1.396 in the underweight group, 1.240 in the non-hypertensive group, and 1.199 in the group with an estimated glomerular filtration rate (eGFR) of less than 30. The mortality impact of ozone, as determined by the administrative district allocation model, was shown to be significant in several groups. Specifically, the impact was 1.159 in individuals under 65 years of age, 1.212 in the BMI < 18.5, 1.160 in the non-diabetic group, and 1.202 in the group with an estimated glomerular filtration rate (eGFR) between 60 and 90 (Fig. 2C). In the context of personal home address allocation, the hazard ratio (HR) for the underweight group was 1.469, for the high blood pressure group was 1.599, and for the group with an estimated glomerular filtration rate (eGFR) of 90 or more was 1.703 HR (Fig. 2D).

Examining the impact of ozone by cause-specific death, O_3_ related death was associated with cardiovascular disease (HR, 1.02; 95% CI, 1.01–1.022), respiratory disease (HR, 1.022; 95% CI, 1.014–1.026), stroke (HR, 1.019; 95% CI, 1.009–1.023), COPD (HR, 1.019; 95% CI, 1.001–1.029), and cancer (HR,1.02; 95% CI, 1.017–1.021). The risk for lung cancer (HR, 1⋅022; 95% CI, 1.016–1.028), and liver cancer (HR, 1.019; 95% CI, 1.012–1.027) were the highest among the cancers (Table 3).

**Table 3:** Cause-specific mortality hazard ratios on ozone concentrations

Cause of death	ICD-10	N	HR	95% CI	
All cause death		6768	1.02	1.016	1.022
Cardiovascular disease	I00-I99	953	1.02	1.016	1.022
Respiratory disease	J00-J99	218	1.022	1.014	1.026
Cancer	C00-C97	1695	1.02	1.017	1.021
Stomach	C16	140	1.018	1.006	1.029
Liver	C22	260	1.019	1.012	1.027
Lung cancer	C34	279	1.022	1.016	1.028
Urinary tract	C64-C68	219	1.02	1.013	1.027
Colorectal cancer	C17-C21	170	1.02	1.010	1.029
Stroke	I20-I22, I24-I25	281	1.019	1.009	1.023
COPD	J44	40	1.019	1.001	1.029

*HR* hazard ratio, *95% CI* 95% confidence intervals, *COPD* chronic obstructive pulmonary disease

## Discussion

In this retrospective cohort study, we demonstrate that long-term exposure to O_3_ increases all-cause and cause-specific mortality, the risk of ESRD in CKD. To adjust effect as cofounder of NO_2_, we used a two-pollutant model. Associations between O_3_ exposure and outcomes were significant after the adjustments for NO_2_. An almost linear exposure–response curve for ozone was previously reported with no threshold or a threshold at very low concentrations. Our result was consistent with previous studies that show the no-threshold linear exposure–response curve [17].

Several studies have been conducted to determine the causal relationship between increased O_3_ exposure and mortality. The effects of short-term exposure to O_3_ on mortality and long-term effect on respiratory diseases have been extensively studied [18–21]. In a meta-analysis of 39 time-series studies, a 0.87% increase in mortality risk was reported for every 10-ppb increase in daily O_3_ concentration at single-day or a 2-day average of lags 0, 1, or 2 days [22]. In a time-series study conducted in 48 cities in the United States, an increase in daily O_3_ concentration of 10-ppb increased the mortality risk by 0.3% (95% CI, 0⋅2–0⋅4) [23]. However, the studies to evaluate the long-term effect of O_3_ have been inconsistent. In a study using data from the American Cancer Society Cancer Prevention Study II (ACCP II) cohort in 2009, associations between O_3_ concentration and the risk of cardiopulmonary death was observed in single-pollutant models; however, only mortality related respiratory disease was associated with O_3_ exposure in two-pollutant models that included O_3_ and PM_2.5_ [24]. On the other hand, another study using the ACCP II cohort in 2016 reported that O_3_ was associated with mortality risk related circulatory disease even in two-pollutant models [25]. These discordant results could be caused by the effect of the interaction of air pollutants showing the concentration–response surface of PM_2.5_ and O_3_ on mortality [6]. In the Canadian Census Health and Environment Cohort (CanCHEC) study included 2.5 million Canadians, researchers reported an increase of non-accidental mortality (HR, 1⋅075; 95% CI: 1⋅067–1⋅084) in a multiple-pollutant model of PM_2.5_, NO_2_, and O_3_, assuming additive associations [1]. Researchers who reported a decreased mortality in increased O_3_ concentrations (HR, 0⋅88; 95% CI, 0⋅82–0⋅96) in 2019, explained that the difference might be due to different source populations, the precision of exposure assessment, as well as the inverse correlation between O_3_ and other harmful pollutants [7]. Many studies have been conducted to evaluate effect of air pollution to renal outcome. The Veterans Administration Normative Aging Study observed an association between increased exposure to PM_2.5_ and decreased renal function [26]. A retrospective cohort study in Hong Kong, including 61,447 participants, observed that PM_2.5_ was associated with increased mortality in CKD [27]. However, few studies have shown the effect of O_3_ on long-term renal outcomes in CKD.

The exact mechanism by which air pollutants affect mortality and kidney disease is unclear. In an animal experiment using cisplatin-induced acute kidney injury model, kidney damage was aggravated by exposure to diesel exhaust particles [28]. The same researchers showed that diesel exhaust particles generated reactive oxidative stress and DNA damage in an adenine-induced CKD animal model [29]. The hypothesis that air pollutants cause deterioration of metabolic factors was also persuasive. Studies have shown a correlation between air pollutants and the carotid intima-media thickness, systolic blood pressure and mean arterial pressure [16, 30] and that exposure to a pollutant activates the hypothalamic–pituitary–adrenal axis and increases glucocorticoid levels [31, 32]. Overall, O_3_ can induce oxidative stress and elevation of inflammatory biomarkers, leading to chronic inflammation in kidney. Hemodynamic, hormonal and metabolic effects of O_3_ may increase the risk of ESRD. Furthermore, considering existing evidence between short-term exposure to elevated O_3_ and increased mortality due to respiratory and circulatory diseases, cause specific mortality in this study supports that there may also be long-term effects through similar mechanisms. It has been hypothesized that the effects of O_3_ may vary depending on gender. The pulmonary response induced by O_3_ could exhibit a sex-specific effect, attributed to variations in airway hyperresponsiveness influenced by factors such as sex hormones and the microbiome [33–35]. The observed difference in the effect of O_3_ according to sex in this study is consistent with previous study. Our study observed a reduced risk of ESRD in participants with hypertension in a model adjusted for comorbidity and socioeconomic factors. However, in sensitivity analysis to predict ESRD, the effect of O_3_ on ESRD in the group of patients with hypertension was not significant, and the effect was maintained in patients without hypertension. This result suggests that there may be a significant interaction between hypertension and O_3_. In vivo, exposure to O_3_ in hypertensive rats has an anti-vasoconstrictive effect by reducing the concentration of serum endothelin-1 [36]. Some researchers have argued that O_3_ could improve hypoxia in patients with peripheral artery disease [37]. Anti-vasoconstrictive effect of O_3_ might be related with the potential to restore renal blood flow, which is reduced in patients with hypertension. Further studies are required to determine whether O_3_ has a protective effect on patients with hypertension in CKD. In the subgroup analysis stratified by BMI, O_3_ showed the highest risk for both ESRD and mortality within the underweight group, with a tendency for these risks to gradually decrease. This observation may be consistent with the concept of the obesity paradox. Air pollutions containing O_3_ could potentially induce malnutrition [38], and malnutrition might serve as an interacting factor by diminishing the capacity to compensate for oxidative stress induced by O_3_ [39]. Although underweight is associated with an increased risk of cardiovascular disease and mortality, the extent to which it increases the risk of ESRD remains a subject of debate. A Taiwan study found that patients with a BMI below 18.5 kg/m^2^ did not experience eGFR decline events in the early or late stages of CKD at non-significantly higher rates than other BMI groups [40]. Studies utilizing the database of the Korean National Health Insurance Service, on the other hand, found that underweight patients had a greater risk of developing ESRD than overweight patients [41]. Loss of body mass exceeding 10% was linked to the most rapid deterioration in renal function. A study conducted on a sample of 9,845,420 individuals aged 20 years or older, who underwent health checkups and were identified from the Korean National Health Insurance Service database, found that being underweight is linked to a higher risk of developing end-stage renal disease [42]. Furthermore, this association becomes stronger as BMI drops. While BMI remains a subject of debate, our data shows that it played a major role when considering its interaction with ozone concentration. Our study findings align with a previous investigation conducted in Korea, which used national health examination data from individuals employed in various workplaces. This prior study demonstrated that individuals classified as underweight were at a significantly elevated risk of developing ESRD. The results revealing a higher risk in early-stage CKD (eGFR > 60), are inconsistent with the previously reported findings. When interpreted in conjunction with the BMI, it is conceivable that a subset of patients with overestimated eGFR may have been included. Given the definition of CKD employed in this study, early-stage CKD patients constitute a cohort characterized by the presence of proteinuria and hematuria. Considering the correlation between air pollutants and proteinuria [43], there is a possibility that there is interaction between O_3_ and kidney disease with proteinuria beyond eGFR. Because we were unable to confirm this association due to insufficient quantitative data for proteinuria, additional research is needed.

The strength of our study is the analysis of the O_3_ measurement data, from a large-scale cohort, with high spatial and temporal resolution. When assessing O_3_ exposure and air pollution concentration at an individual level, we observed an association between a model that applied the average value of the measured data from the monitoring sites, per each administrative district based on the patients’ residence, and another model that used IDW interpolation and assigned it to the coordinate point of the residence. This study had several limitations. First, because we used the residential zip codes rather than the exact house address or place of death of each participant to determine exposure level, we expect some degree of measurement error. Second, most of the participants’ residences were situated in a specific metropolitan area, which may lead to selection bias. However, since the majority of the population in South Korea resides in this area, we believe that the exposure detected in this population is a good representation of the exposure detected in our cohort. Third, the most recent data used in this study is four years old; therefore, it is uncertain whether exposures and outcomes will be similar to that of current data. Fourth, there was limited direct usage in model fitting due to the lack of information in our data that might correct the lifestyle choices and health condition of CKD patients; however, factors pertaining to regional characteristics were adjusted.

## Conclusions

In conclusion, based on a large cohort of participants with CKD, long-term exposure to O_3_ is associated with an increased risk of ESRD and mortality. Our findings highlight the need for better measures to control O_3_ exposure and the emission of pollutants that contribute to the increase of O_3_ in the atmosphere.

### Supplementary Information

**Supplementary Material 1.**

## Data Availability

The datasets generated during and/or analyzed during the current study are available from the corresponding author on reasonable request.

## Abbreviations

CI: Confidence interval
CKD: Chronic kidney disease
CO: Carbon monoxide
COPD: Chronic obstructive pulmonary disease
CVD: Cardiovascular disease
DM: Diabetes mellitus
eGFR: Estimated glomerular filtration rate
ESRD: End stage renal disease
GIS: Geographic Information System
HR: Hazard ratio
HTN: Hypertension
IDW: Inverse-distance weighting
MDRD: Modification of diet in the renal disease equation
NO_2_: Nitrogen dioxide
O_3_: Ozone
PM_2.5_: Particulate matter
ppb: Parts per billion
SO_2_: Sulfur dioxide
