# Supplementary material for: Long-term ozone exposure and mortality in patients with chronic kidney disease: a large cohort study
Source: BMC Nephrol. 2024 Feb 28;25:74. doi: 10.1186/s12882-024-03500-6 (PMC10900590; doi:10.1186/s12882-024-03500-6)
Supplement: Supplementary file 1 — Supplementary Material 1. [file 12882_2024_3500_MOESM1_ESM.docx]

**Long-term ozone exposure and mortality in patients with chronic kidney disease: a large cohort study**

**Author**

Ejin Kim^1¶^, Hyuk Huh^2¶^, Yongwon Mo^3^, Jae Yoon Park^4^, Jiyun Jung^5^, Hajeong Lee^6^, Sejoong Kim^10,12^, Dong Ki Kim^6,7,12^, Yon Su Kim^6,7,12^, Chun Soo Lim^9,12^, Jung Pyo Lee^9,12^, Yong Chul Kim^6*,^ Ho Kim^1,11*^

**Affiliation**

^1^Institute of Health and Environment and Graduate School of Public Health, Seoul National University, Republic of Korea

^2^Department of Internal Medicine, Inje University Busan Paik Hospital, Busan, Republic of Korea

^3^Department of Landscape architecture, Yeungnam University Gyeongsan, Republic of Korea

^4^Department of Internal Medicine, Dongguk University Ilsan Hospital, Gyeonggi-do, Republic of Korea

^5^Data Management and Statistics Institute, Dongguk University Ilsan Hospital, Ilsan, Republic of Korea

^6^Department of Internal Medicine, Seoul National University Hospital, Seoul, Republic of Korea

^7^Kidney Research Institute, Seoul National University Hospital, Republic of Korea

^8^Department of Medical Science, Seoul National University College of Medicine, Seoul, Republic of Korea

^9^Department of Internal Medicine, Seoul National University Boramae Medical Center, Seoul, Republic of Korea

^10^Department of Internal Medicine, Seoul National University Bundang Hospital, Seongnam, Republic of Korea

^11^Department of Biostatistics and Epidemiology, School of Public Health, Seoul National University, Seoul, Republic of Korea

^12^Department of Internal Medicine, Seoul National University College of Medicine, Seoul, Republic of Korea

^¶^EK and HH contributed equally to this research.

*Corresponding author

Yong Chul Kim, MD, PhD

Associate Professor

Department of Internal Medicine, Seoul National University Hospital

101, Daehak-ro, Jongno-gu, Seoul, South Korea

Tel: 82-02-2072-4072

E-mail: imyongkim@gmail.com

Ho Kim, PhD

Professor

Graduate School of Public Health, Seoul National University,

Room 708, Building 220, Graduate School of Public Health, Seoul National University, Gwanak-Ro Gwanak-Gu, Seoul 08826, Republic of Korea,

E-mail: [hokim@snu.ac.kr](mailto:hokim@snu.ac.kr)

^*^YCK and HK contributed equally to this research as co-corresponding author.

**Supplementary Materials**

**Figure S1. Analyses of risk of renal outcomes, end stage renal disease (ESRD) and death by ozone concentrations**


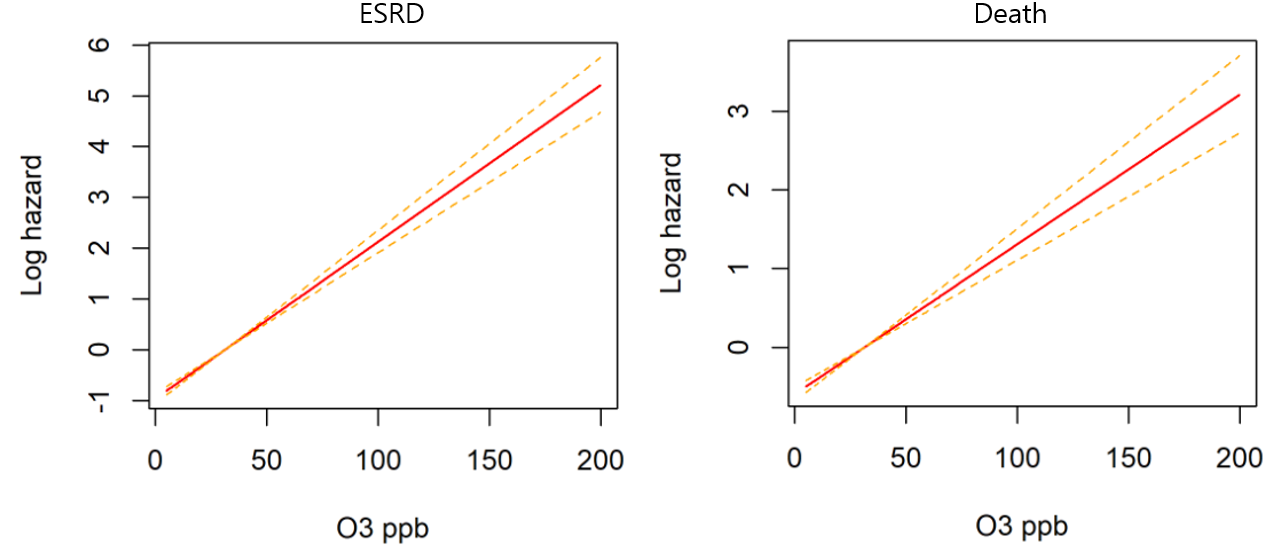


**Table S1.** Association of annual mean maximum daily 8-hour O3 concentrations from the previous year (1-year rolling average) with ESRD and all-cause mortality in participants with CKD excluding CKD stage 4 (eGFR<30 mL/min/1.73 m²)

**Table S2.** Subgroup analysis of the association between ozone concentration and end-stage renal disease, all-cause mortality by sex, age, BMI, diagnosis with hypertension, diabetic mellitus and CKD stages

**Table S1.** Association of annual mean maximum daily 8-hour O3 concentrations from the previous year (1-year rolling average) with ESRD and all-cause mortality in participants with CKD excluding CKD stage 4 (eGFR<30 mL/min/1.73 m²)

| Outcomes | End-stage renal disease (ESRD) | | | | | | All-cause Morality | | | | | |
| --- | --- | --- | --- | --- | --- | --- | --- | --- | --- | --- | --- | --- |
| Exposure assessments | Districts | | | Points | | | Districts | | | Points | | |
|  | HR | 95% CI | | HR | 95% CI | | HR | 95% CI | | HR | 95% CI | |
| MODEL |  |  |  |  |  |  |  |  |  |  |  |  |
| Age | 0.997 | 0.994 | 0.999 | 0.996 | 0.992 | 1.001 | 1.072 | 1.07 | 1.075 | 1.064 | 1.06 | 1.067 |
| Male | 2.016 | 1.857 | 2.189 | 1.852 | 1.595 | 2.151 | 1.988 | 1.881 | 2.101 | 2.151 | 1.965 | 2.355 |
| Hypertension | 1.211 | 1.112 | 1.318 | 1.231 | 1.036 | 1.463 | 1.192 | 1.126 | 1.261 | 1.646 | 1.501 | 1.805 |
| Diabetic Mellitus | 3.84 | 3.531 | 4.175 | 3.717 | 3.189 | 4.331 | 1.373 | 1.299 | 1.451 | 1.301 | 1.189 | 1.423 |
| BMI<18.5 | 0.216 | 0.19 | 0.244 | 0.294 | 0.234 | 0.369 | 0.708 | 0.664 | 0.754 | 0.83 | 0.749 | 0.921 |
| 23≤BMI<25 | 0.602 | 0.54 | 0.67 | 0.719 | 0.59 | 0.875 | 0.596 | 0.55 | 0.645 | 0.566 | 0.499 | 0.642 |
| 25$\leq$BMI | 0.539 | 0.49 | 0.594 | 0.649 | 0.542 | 0.776 | 0.518 | 0.481 | 0.559 | 0.41 | 0.361 | 0.466 |
| O3 (ppb) | **1.043** | **1.036** | **1.05** | **1.014** | **1** | **1.029** | **1.033** | **1.029** | **1.037** | **1.063** | **1.054** | **1.072** |
| MODEL1 |  |  |  |  |  |  |  |  |  |  |  |  |
| Age | 0.971 | 0.968 | 0.974 | 0.968 | 0.964 | 0.973 | 1.061 | 1.059 | 1.064 | 1.054 | 1.05 | 1.058 |
| Male | 1.506 | 1.386 | 1.635 | 1.476 | 1.27 | 1.714 | 1.785 | 1.688 | 1.887 | 1.992 | 1.818 | 2.182 |
| Hypertension | 0.991 | 0.91 | 1.079 | 1.008 | 0.849 | 1.197 | 1.116 | 1.055 | 1.182 | 1.574 | 1.436 | 1.726 |
| Diabetic Mellitus | 2.977 | 2.739 | 3.235 | 2.885 | 2.478 | 3.358 | 1.275 | 1.206 | 1.348 | 1.232 | 1.126 | 1.348 |
| BMI<18.5 | 0.313 | 0.276 | 0.355 | 0.369 | 0.294 | 0.464 | 0.767 | 0.719 | 0.818 | 0.865 | 0.78 | 0.96 |
| 23≤BMI<25 | 0.662 | 0.595 | 0.738 | 0.703 | 0.577 | 0.856 | 0.598 | 0.553 | 0.648 | 0.557 | 0.491 | 0.631 |
| 25$\leq$BMI | 0.569 | 0.517 | 0.627 | 0.634 | 0.53 | 0.759 | 0.517 | 0.48 | 0.558 | 0.409 | 0.36 | 0.464 |
| e-GFR (mL/min/1.73 m²) | 0.93 | 0.927 | 0.932 | 0.925 | 0.92 | 0.93 | 0.977 | 0.975 | 0.979 | 0.981 | 0.979 | 0.984 |
| O3 (ppb) | **1.033** | **1.025** | **1.04** | 0.995 | 0.981 | 1.01 | **1.029** | **1.025** | **1.034** | **1.058** | **1.049** | **1.068** |
| MODEL2 |  |  |  |  |  |  |  |  |  |  |  |  |
| Age | 0.97 | 0.967 | 0.973 | 0.968 | 0.962 | 0.973 | 1.062 | 1.059 | 1.064 | 1.053 | 1.049 | 1.057 |
| Male | 1.559 | 1.428 | 1.703 | 1.454 | 1.226 | 1.724 | 1.749 | 1.65 | 1.854 | 1.955 | 1.774 | 2.154 |
| Hypertension | 1.001 | 0.917 | 1.094 | 1.077 | 0.897 | 1.292 | 1.066 | 1.006 | 1.13 | 1.545 | 1.404 | 1.699 |
| Diabetic Mellitus | 3.105 | 2.841 | 3.394 | 3.303 | 2.772 | 3.936 | 1.265 | 1.194 | 1.34 | 1.187 | 1.078 | 1.306 |
| BMI<18.5 | 0.334 | 0.293 | 0.382 | 0.436 | 0.338 | 0.562 | 0.741 | 0.692 | 0.792 | 0.843 | 0.754 | 0.942 |
| 23≤BMI<25 | 0.652 | 0.582 | 0.731 | 0.685 | 0.547 | 0.858 | 0.593 | 0.547 | 0.643 | 0.568 | 0.498 | 0.647 |
| 25$\leq$BMI | 0.53 | 0.479 | 0.587 | 0.564 | 0.459 | 0.693 | 0.51 | 0.472 | 0.551 | 0.399 | 0.349 | 0.457 |
| e-GFR (mL/min/1.73 m²) | 0.931 | 0.928 | 0.934 | 0.925 | 0.919 | 0.931 | 0.977 | 0.976 | 0.979 | 0.983 | 0.98 | 0.986 |
| O3 (ppb) | **1.061** | **1.05** | **1.071** | 1.014 | 0.994 | 1.034 | **1.027** | **1.021** | **1.033** | **1.062** | **1.05** | **1.074** |
| NO2(ppb) | 1.018 | 1.011 | 1.026 | 0.987 | 0.97 | 1.004 | 1.01 | 1.005 | 1.015 | 1.022 | 1.012 | 1.031 |
| MODEL3 |  |  |  |  |  |  |  |  |  |  |  |  |
| Age | 0.97 | 0.968 | 0.973 | 0.967 | 0.962 | 0.973 | 1.062 | 1.059 | 1.064 | 1.054 | 1.05 | 1.058 |
| Male | 1.558 | 1.426 | 1.703 | 1.46 | 1.231 | 1.731 | 1.752 | 1.653 | 1.857 | 1.964 | 1.782 | 2.164 |
| Hypertension | 0.988 | 0.903 | 1.081 | 1.09 | 0.907 | 1.309 | 1.08 | 1.018 | 1.145 | 1.547 | 1.406 | 1.702 |
| Diabetic Mellitus | 3.106 | 2.841 | 3.396 | 3.3 | 2.769 | 3.933 | 1.265 | 1.193 | 1.34 | 1.186 | 1.078 | 1.306 |
| BMI<18.5 | 0.337 | 0.295 | 0.385 | 0.438 | 0.339 | 0.564 | 0.737 | 0.688 | 0.788 | 0.843 | 0.754 | 0.941 |
| 23≤BMI<25 | 0.649 | 0.578 | 0.728 | 0.686 | 0.548 | 0.859 | 0.59 | 0.544 | 0.64 | 0.571 | 0.501 | 0.651 |
| 25$\leq$BMI | 0.528 | 0.477 | 0.585 | 0.564 | 0.46 | 0.693 | 0.512 | 0.474 | 0.553 | 0.402 | 0.351 | 0.46 |
| e-GFR (mL/min/1.73 m²) | 0.931 | 0.928 | 0.934 | 0.925 | 0.919 | 0.931 | 0.977 | 0.975 | 0.979 | 0.983 | 0.98 | 0.986 |
| O3 (ppb) | **1.063** | **1.052** | **1.074** | 1.015 | 0.994 | 1.036 | **1.023** | **1.017** | **1.03** | **1.053** | **1.041** | **1.065** |
| NO2(ppb) | 1.018 | 1.01 | 1.027 | 0.987 | 0.969 | 1.005 | 1.01 | 1.005 | 1.015 | 1.029 | 1.019 | 1.039 |
| Beds in medical facilities per 1,000 people | 1.009 | 0.995 | 1.024 | 1.016 | 0.993 | 1.04 | 0.984 | 0.976 | 0.993 | 0.992 | 0.979 | 1.005 |
| Financial independence | 1.003 | 1 | 1.007 | 0.999 | 0.993 | 1.005 | 0.997 | 0.995 | 0.999 | 0.994 | 0.991 | 0.998 |

**Table S2.** Subgroup analysis of the association between ozone concentration and end-stage renal disease, all-cause mortality by sex, age, BMI, diagnosis with hypertension, diabetic mellitus and CKD stages

|  | End-stage renal disease (ESRD) | | | | | | All-cause mortality | | | | | |
| --- | --- | --- | --- | --- | --- | --- | --- | --- | --- | --- | --- | --- |
|  | Districts | | | Points | | | Districts | | | Points | | |
| Parameter | HR | 95% CI | | HR | 95% CI | | HR | 95% CI | | HR | 95% CI | |
| **Sex** |  |  |  |  |  |  |  |  |  |  |  |  |
| Age | 0.987 | 0.986 | 0.989 | 0.987 | 0.984 | 0.989 | 1.06 | 1.058 | 1.062 | 1.051 | 1.048 | 1.054 |
| Hypertension | 0.927 | 0.884 | 0.972 | 0.786 | 0.712 | 0.867 | 1.074 | 1.027 | 1.124 | 1.538 | 1.428 | 1.657 |
| Diabetic Mellitus | 1.675 | 1.601 | 1.751 | 1.676 | 1.538 | 1.827 | 1.268 | 1.214 | 1.324 | 1.168 | 1.084 | 1.257 |
| BMI<18.5 | 0.493 | 0.462 | 0.527 | 0.451 | 0.396 | 0.514 | 0.912 | 0.866 | 0.961 | 1.014 | 0.929 | 1.106 |
| 23≤BMI<25 | 0.845 | 0.796 | 0.896 | 0.892 | 0.794 | 1.003 | 0.662 | 0.622 | 0.705 | 0.633 | 0.57 | 0.703 |
| 25$\leq$BMI | 0.779 | 0.738 | 0.822 | 0.865 | 0.778 | 0.961 | 0.601 | 0.566 | 0.638 | 0.461 | 0.414 | 0.513 |
| e-GFR (mL/min/1.73 m²) | 0.927 | 0.926 | 0.928 | 0.922 | 0.92 | 0.925 | 0.972 | 0.971 | 0.973 | 0.975 | 0.973 | 0.977 |
| NO2(ppb) | 1.016 | 1.012 | 1.02 | 0.991 | 0.982 | 1 | 1.004 | 1 | 1.008 | 1.025 | 1.018 | 1.033 |
| Number of medical institution beds per 1,000 people | 1.003 | 0.997 | 1.01 | 1.012 | 1 | 1.025 | 0.979 | 0.973 | 0.986 | 0.992 | 0.982 | 1.002 |
| Financial independence | 1.001 | 1 | 1.003 | 1 | 0.997 | 1.003 | 0.999 | 0.998 | 1.001 | 0.995 | 0.992 | 0.998 |
| Sex(Male) | 1.428 | 1.089 | 1.872 | 1.066 | 0.622 | 1.829 | 1.953 | 1.53 | 2.495 | 1.828 | 1.147 | 2.915 |
| O3(ppb) | 1.052 | 1.045 | 1.06 | 1.023 | 1.008 | 1.037 | 1.017 | 1.01 | 1.023 | 1.042 | 1.029 | 1.056 |
| O3(ppb)*Sex | 0.995 | 0.987 | 1.003 | 1.004 | 0.986 | 1.022 | 0.993 | 0.986 | 1.001 | 0.997 | 0.982 | 1.013 |
| **Age group** |  |  |  |  |  |  |  |  |  |  |  |  |
| Male | 1.222 | 1.17 | 1.276 | 1.2 | 1.104 | 1.305 | 1.633 | 1.565 | 1.705 | 1.688 | 1.569 | 1.816 |
| Hypertension | 0.927 | 0.884 | 0.972 | 0.782 | 0.708 | 0.862 | 1.136 | 1.086 | 1.188 | 1.657 | 1.538 | 1.785 |
| Diabetic Mellitus | 1.651 | 1.579 | 1.726 | 1.635 | 1.501 | 1.78 | 1.274 | 1.219 | 1.331 | 1.183 | 1.098 | 1.275 |
| BMI<18.5 | 0.491 | 0.46 | 0.524 | 0.453 | 0.398 | 0.516 | 0.927 | 0.88 | 0.977 | 1.014 | 0.929 | 1.107 |
| 23≤BMI<25 | 0.838 | 0.79 | 0.89 | 0.88 | 0.783 | 0.989 | 0.658 | 0.618 | 0.7 | 0.648 | 0.583 | 0.719 |
| 25$\leq$BMI | 0.779 | 0.738 | 0.823 | 0.861 | 0.775 | 0.957 | 0.569 | 0.536 | 0.604 | 0.451 | 0.405 | 0.502 |
| e-GFR (mL/min/1.73 m²) | 0.927 | 0.926 | 0.928 | 0.922 | 0.92 | 0.925 | 0.971 | 0.97 | 0.972 | 0.974 | 0.972 | 0.975 |
| NO2(ppb) | 1.016 | 1.011 | 1.02 | 0.992 | 0.983 | 1.001 | 1.008 | 1.004 | 1.012 | 1.021 | 1.014 | 1.029 |
| Number of medical institution beds per 1,000 people | 1.002 | 0.995 | 1.008 | 1.01 | 0.998 | 1.023 | 0.985 | 0.979 | 0.992 | 1.001 | 0.991 | 1.012 |
| Financial independence | 1.002 | 1 | 1.003 | 1 | 0.997 | 1.003 | 0.997 | 0.995 | 0.998 | 0.995 | 0.992 | 0.998 |
| Age≥65 | 1.176 | 0.898 | 1.54 | 1.137 | 0.66 | 1.957 | 6.788 | 4.65 | 9.91 | 3.3 | 1.866 | 5.834 |
| O3(ppb) | 1.059 | 1.052 | 1.066 | 1.036 | 1.022 | 1.05 | 1.019 | 1.007 | 1.031 | 1.032 | 1.014 | 1.05 |
| O3(ppb)* Age≥65 | 0.984 | 0.976 | 0.993 | 0.985 | 0.967 | 1.002 | 0.986 | 0.975 | 0.998 | 0.998 | 0.979 | 1.016 |
| **BMI** |  |  |  |  |  |  |  |  |  |  |  |  |
| Age | 0.987 | 0.986 | 0.989 | 0.987 | 0.984 | 0.989 | 1.06 | 1.058 | 1.062 | 1.051 | 1.048 | 1.054 |
| Male | 1.221 | 1.169 | 1.274 | 1.195 | 1.099 | 1.299 | 1.594 | 1.527 | 1.664 | 1.681 | 1.562 | 1.808 |
| Hypertension | 0.926 | 0.883 | 0.971 | 0.786 | 0.713 | 0.868 | 1.076 | 1.028 | 1.125 | 1.025 | 1.017 | 1.033 |
| Diabetic Mellitus | 1.676 | 1.603 | 1.753 | 1.679 | 1.541 | 1.83 | 1.266 | 1.212 | 1.322 | 0.992 | 0.982 | 1.002 |
| e-GFR (mL/min/1.73 m²) | 0.927 | 0.926 | 0.928 | 0.27 | 0.121 | 0.604 | 0.972 | 0.971 | 0.973 | 1.54 | 1.429 | 1.659 |
| NO2(ppb) | 1.016 | 1.012 | 1.02 | 1.037 | 0.476 | 2.262 | 1.004 | 1 | 1.008 | 1.165 | 1.082 | 1.255 |
| Number of medical institution beds per 1,000 people | 1.003 | 0.997 | 1.01 | 1.796 | 0.917 | 3.517 | 0.979 | 0.973 | 0.985 | 0.975 | 0.973 | 0.977 |
| Financial independence | 1.001 | 1 | 1.003 | 0.922 | 0.92 | 0.925 | 0.999 | 0.998 | 1.001 | 0.995 | 0.992 | 0.998 |
| BMI<18.5 | 0.336 | 0.23 | 0.493 | 0.991 | 0.982 | 1 | 0.575 | 0.432 | 0.765 | 0.728 | 0.424 | 1.25 |
| 23≤BMI<25 | 0.983 | 0.672 | 1.437 | 1.013 | 1.001 | 1.026 | 0.889 | 0.617 | 1.28 | 1.077 | 0.541 | 2.147 |
| 25$\leq$BMI | 1.022 | 0.722 | 1.446 | 1 | 0.996 | 1.003 | 0.647 | 0.456 | 0.918 | 1.014 | 0.505 | 2.036 |
| O3(ppb) | 1.05 | 1.043 | 1.057 | 1.029 | 1.014 | 1.045 | 1.009 | 1.002 | 1.016 | 1.043 | 1.03 | 1.057 |
| O3(ppb)* BMI<18.5 | 1.012 | 1 | 1.024 | 1.018 | 0.991 | 1.046 | 1.015 | 1.006 | 1.025 | 1.011 | 0.993 | 1.03 |
| O3(ppb)* 23≤BMI<25 | 0.995 | 0.984 | 1.007 | 0.995 | 0.97 | 1.021 | 0.99 | 0.979 | 1.002 | 0.982 | 0.96 | 1.005 |
| O3(ppb)* 25$\leq$BMI | 0.992 | 0.981 | 1.002 | 0.976 | 0.955 | 0.998 | 0.998 | 0.987 | 1.009 | 0.974 | 0.952 | 0.997 |
| **Hypertension** |  |  |  |  |  |  |  |  |  |  |  |  |
| Age | 0.987 | 0.986 | 0.989 | 0.987 | 0.984 | 0.989 | 1.06 | 1.058 | 1.062 | 1.051 | 1.048 | 1.054 |
| Male | 1.219 | 1.168 | 1.273 | 1.191 | 1.096 | 1.295 | 1.594 | 1.527 | 1.663 | 1.679 | 1.56 | 1.806 |
| Diabetic Mellitus | 1.677 | 1.603 | 1.754 | 1.676 | 1.538 | 1.826 | 1.269 | 1.215 | 1.325 | 1.167 | 1.083 | 1.256 |
| BMI<18.5 | 0.493 | 0.462 | 0.526 | 0.454 | 0.398 | 0.517 | 0.912 | 0.865 | 0.961 | 1.012 | 0.928 | 1.104 |
| 23≤BMI<25 | 0.843 | 0.795 | 0.895 | 0.892 | 0.794 | 1.003 | 0.662 | 0.622 | 0.705 | 0.634 | 0.571 | 0.704 |
| 25$\leq$BMI | 0.778 | 0.737 | 0.822 | 0.866 | 0.779 | 0.962 | 0.601 | 0.566 | 0.638 | 0.46 | 0.413 | 0.512 |
| e-GFR (mL/min/1.73 m²) | 0.927 | 0.926 | 0.928 | 0.922 | 0.92 | 0.925 | 0.972 | 0.971 | 0.973 | 0.975 | 0.973 | 0.976 |
| NO2(ppb) | 1.016 | 1.012 | 1.02 | 0.991 | 0.982 | 1 | 1.004 | 1 | 1.008 | 1.026 | 1.018 | 1.033 |
| Number of medical institution beds per 1,000 people | 1.003 | 0.997 | 1.01 | 1.013 | 1 | 1.025 | 0.979 | 0.973 | 0.985 | 0.992 | 0.981 | 1.002 |
| Financial independence | 1.001 | 1 | 1.003 | 1 | 0.996 | 1.003 | 0.999 | 0.998 | 1.001 | 0.995 | 0.992 | 0.998 |
| Hypertension | 1.12 | 0.818 | 1.534 | 1.648 | 0.868 | 3.129 | 1.216 | 0.935 | 1.582 | 0.458 | 0.282 | 0.744 |
| O3(ppb) | 1.051 | 1.045 | 1.056 | 1.031 | 1.019 | 1.042 | 1.013 | 1.008 | 1.019 | 1.026 | 1.015 | 1.036 |
| O3(ppb)* Hypertension | 0.994 | 0.984 | 1.004 | 0.976 | 0.956 | 0.997 | 0.996 | 0.988 | 1.004 | 1.041 | 1.025 | 1.058 |
| **Diabetic Mellitus** |  |  |  |  |  |  |  |  |  |  |  |  |
| Age | 0.987 | 0.986 | 0.989 | 0.987 | 0.984 | 0.99 | 1.06 | 1.058 | 1.062 | 1.051 | 1.048 | 1.054 |
| Male | 1.217 | 1.166 | 1.271 | 1.189 | 1.094 | 1.293 | 1.591 | 1.525 | 1.661 | 1.675 | 1.557 | 1.802 |
| Hypertension | 0.927 | 0.884 | 0.972 | 0.787 | 0.713 | 0.868 | 1.077 | 1.029 | 1.126 | 1.539 | 1.429 | 1.659 |
| BMI<18.5 | 0.494 | 0.463 | 0.527 | 0.453 | 0.397 | 0.516 | 0.915 | 0.869 | 0.965 | 1.015 | 0.931 | 1.108 |
| 23≤BMI<25 | 0.844 | 0.795 | 0.896 | 0.893 | 0.794 | 1.004 | 0.662 | 0.622 | 0.705 | 0.632 | 0.569 | 0.702 |
| 25$\leq$BMI | 0.779 | 0.737 | 0.822 | 0.867 | 0.78 | 0.964 | 0.601 | 0.566 | 0.638 | 0.461 | 0.414 | 0.513 |
| e-GFR (mL/min/1.73 m²) | 0.927 | 0.926 | 0.928 | 0.922 | 0.92 | 0.925 | 0.972 | 0.971 | 0.973 | 0.975 | 0.973 | 0.977 |
| NO2(ppb) | 1.016 | 1.012 | 1.02 | 0.991 | 0.982 | 1 | 1.004 | 1 | 1.008 | 1.025 | 1.018 | 1.033 |
| Number of medical institution beds per 1,000 people | 1.003 | 0.997 | 1.01 | 1.013 | 1 | 1.025 | 0.979 | 0.973 | 0.986 | 0.992 | 0.982 | 1.002 |
| Financial independence | 1.001 | 1 | 1.003 | 1 | 0.997 | 1.003 | 0.999 | 0.998 | 1.001 | 0.995 | 0.992 | 0.998 |
| Diabetic Mellitus | 2.217 | 1.694 | 2.903 | 2.352 | 1.371 | 4.033 | 2.098 | 1.649 | 2.668 | 1.797 | 1.132 | 2.851 |
| O3(ppb) | 1.053 | 1.047 | 1.06 | 1.03 | 1.017 | 1.044 | 1.019 | 1.014 | 1.025 | 1.046 | 1.035 | 1.058 |
| O3(ppb)* Diabetic Mellitus | 0.991 | 0.983 | 1 | 0.989 | 0.971 | 1.006 | 0.984 | 0.976 | 0.991 | 0.986 | 0.97 | 1.001 |
| **CKD stage** |  |  |  |  |  |  |  |  |  |  |  |  |
| AGE | 0.983 | 0.982 | 0.985 | 0.981 | 0.978 | 0.984 | 1.061 | 1.059 | 1.063 | 1.052 | 1.049 | 1.055 |
| Male | 1.262 | 1.209 | 1.318 | 1.271 | 1.169 | 1.382 | 1.617 | 1.549 | 1.689 | 1.704 | 1.583 | 1.835 |
| Hypertension | 0.862 | 0.822 | 0.904 | 0.731 | 0.662 | 0.806 | 1.063 | 1.016 | 1.112 | 1.514 | 1.405 | 1.631 |
| Diabetic Mellitus | 1.674 | 1.601 | 1.751 | 1.679 | 1.54 | 1.83 | 1.283 | 1.229 | 1.34 | 1.171 | 1.088 | 1.261 |
| BMI<18.5 | 0.447 | 0.419 | 0.477 | 0.417 | 0.366 | 0.474 | 0.891 | 0.845 | 0.938 | 1.009 | 0.925 | 1.101 |
| 23≤BMI<25 | 0.778 | 0.734 | 0.826 | 0.808 | 0.719 | 0.908 | 0.656 | 0.616 | 0.698 | 0.637 | 0.574 | 0.708 |
| 25$\leq$BMI | 0.714 | 0.676 | 0.754 | 0.749 | 0.674 | 0.831 | 0.596 | 0.561 | 0.633 | 0.464 | 0.417 | 0.516 |
| NO2(ppb) | 1.015 | 1.011 | 1.019 | 0.99 | 0.981 | 0.999 | 1.004 | 1 | 1.008 | 1.026 | 1.018 | 1.034 |
| Number of medical institution beds per 1,000 people | 1.006 | 1 | 1.013 | 1.017 | 1.005 | 1.03 | 0.979 | 0.973 | 0.986 | 0.992 | 0.982 | 1.003 |
| Financial independence | 1 | 0.999 | 1.002 | 0.998 | 0.995 | 1.001 | 0.999 | 0.998 | 1.001 | 0.995 | 0.992 | 0.998 |
| e-GFR<30(mL/min/1.73 m²) | 76.218 | 17.324 | 335.32 | 295.861 | 2.746 | 31872.15 | 10.614 | 4.607 | 24.453 | 15.08 | 3.598 | 63.206 |
| 30≤e-GFR<60 | 13.936 | 3.112 | 62.407 | 109.306 | 0.993 | 12031.8 | 2.172 | 0.94 | 5.015 | 4.021 | 0.957 | 16.896 |
| 60≤e-GFR<90 | 0.541 | 0.105 | 2.781 | 1.83 | 0.014 | 240.647 | 0.86 | 0.361 | 2.047 | 1.014 | 0.227 | 4.531 |
| O3(ppb) | 1.029 | 0.982 | 1.078 | 1.019 | 0.874 | 1.187 | 1.024 | 0.998 | 1.05 | 1.077 | 1.03 | 1.126 |
| O3(ppb)*CKD stage4(e-GFR<30) | 1.017 | 0.971 | 1.066 | 1.007 | 0.864 | 1.174 | 0.974 | 0.949 | 1 | 0.953 | 0.911 | 0.998 |
| O3(ppb)*CKD stage3(30≤e-GFR<60) | 1.005 | 0.958 | 1.053 | 0.965 | 0.827 | 1.126 | 0.998 | 0.972 | 1.025 | 0.969 | 0.926 | 1.015 |
| O3(ppb)*CKD stage2(60≤e-GFR<90) | 1.035 | 0.983 | 1.09 | 1.029 | 0.877 | 1.208 | 1 | 0.973 | 1.028 | 0.989 | 0.942 | 1.038 |

ESRD, end stage renal disease; O_3_, ozone; NO_2_, nitrogen dioxide; HR, hazard ratio; 95% CI, 95% confidence intervals
